# Therapeutic targeting of p90 ribosomal S6 kinase

**DOI:** 10.3389/fcell.2023.1297292

**Published:** 2023-12-19

**Authors:** Eric B. Wright, Deborah A. Lannigan

**Affiliations:** ^1^ Department Biomedical Engineering, Vanderbilt University, Nashville, TN, United States; ^2^ Department Pathology, Vanderbilt University Medical Center, Nashville, TN, United States

**Keywords:** p90 ribosomal S6 kinase, RSK, small molecule inhibitor, substrate, phosphorylation, p90RSK

## Abstract

The Serine/Threonine protein kinase family, p90 ribosomal S6 kinases (RSK) are downstream effectors of extracellular signal regulated kinase 1/2 (ERK1/2) and are activated in response to tyrosine kinase receptor or G-protein coupled receptor signaling. RSK contains two distinct kinase domains, an N-terminal kinase (NTKD) and a C-terminal kinase (CTKD). The sole function of the CTKD is to aid in the activation of the NTKD, which is responsible for substrate phosphorylation. RSK regulates various homeostatic processes including those involved in transcription, translation and ribosome biogenesis, proliferation and survival, cytoskeleton, nutrient sensing, excitation and inflammation. RSK also acts as a major negative regulator of ERK1/2 signaling. RSK is associated with numerous cancers and has been primarily studied in the context of transformation and metastasis. The development of specific RSK inhibitors as cancer therapeutics has lagged behind that of other members of the mitogen-activated protein kinase signaling pathway. Importantly, a pan-RSK inhibitor, PMD-026, is currently in phase I/1b clinical trials for metastatic breast cancer. However, there are four members of the RSK family, which have overlapping and distinct functions that can vary in a tissue specific manner. Thus, a problem for transitioning a RSK inhibitor to the clinic may be the necessity to develop isoform specific inhibitors, which will be challenging as the NTKDs are very similar to each other. CTKD inhibitors have limited use as therapeutics as they are not able to inhibit the activity of the NTKD but could be used in the development of proteolysis-targeting chimeras.

## 1 Introduction

**FIGURE 1:**
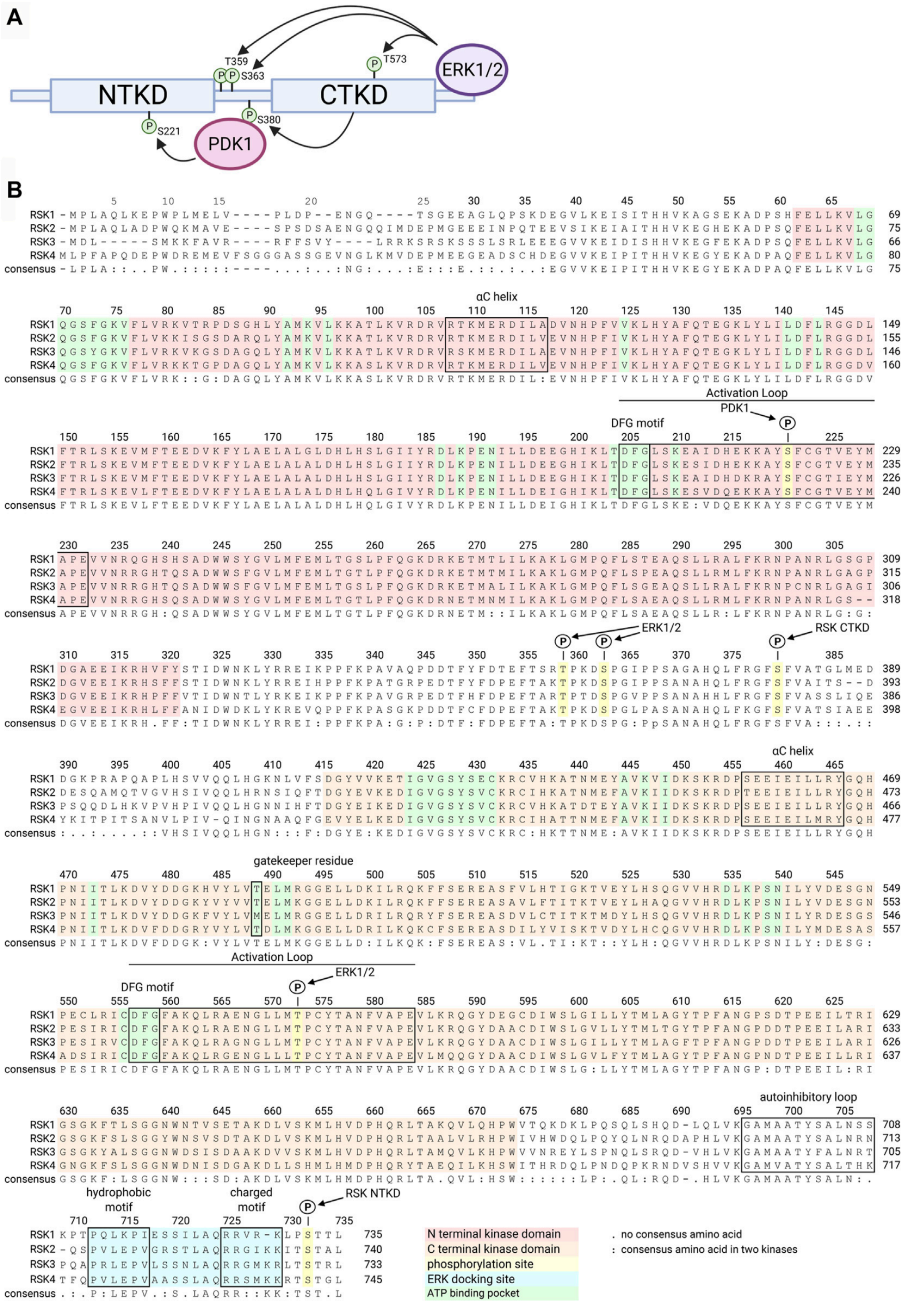
RSK structure family sequence alignment. Numbering is relative to human RSK1. A schematic of RSK shows the NTKD and CTKD with phosphorylation sites required to activate the RSK NTKD, which then phosphorylates RSK substrates **(A)**. ERK1/2 docks on the C terminus and phosphorylates several sites within the CTKD and linker region. CTKD phosphorylation of Ser380 within the linker region creates a docking site for PDK1, which then phosphorylates Ser221 in the NTKD, resulting in full activation of RSK. The 4 isoforms of the human RSK family are highly homologous, as shown in the consensus sequence **(B)**. Key functional regions of the kinases are highlighted or annotated.

The family of p90 ribosomal S6 kinases (RSK), also referred to as mitogen-activated protein kinase -activated protein kinase (MAPKAP-K1), belong to the Serine/Threonine protein kinase family. In vertebrates there are four family members: RSK1 (HGMW-approved symbol RPS6KA1), RSK2 (RPS6KA3), RSK3 (RPS6KA2), and RSK4 (RPS6KA6). Phylogenetic analysis indicates that RSK2 and RSK4 are more closely related than to RSK3 with RSK1 being the most distant relative (Ludwik and Lannigan, 2016) (Figure 1). RSK is activated in response to activation of the mitogen activated protein-kinase (MAPK) pathway through tyrosine kinase receptor or G-protein coupled receptor signaling (Lannigan, 2022). Activation of RSK1, RSK2, and RSK3 is complex and requires inputs from extracellular signal regulated kinase 1/2 (ERK1/2) and 3-phosphinositide dependent protein kinase-1 (PDK1) (Sturgill et al., 1988; Jensen et al., 1999) (Figure 1A). In contrast, RSK4 has high basal activity and does not appear to require PDK1 for activation (Dummler et al., 2005). The RSK family members are composed of two distinct kinase domains with the N-terminal kinase domain (NTKD) being related to the protein kinase A, G, and C subfamily of kinases and the CTKD being related to the Ca2+/calmodulin-dependent subfamily of kinases (Fisher and Blenis, 1996). The NTKD is responsible for substrate phosphorylation whereas the only known function for the CTKD is autophosphorylation. Based on molecular studies the extreme C-terminus of RSK2 is known to act as an inhibitor of the CTKD and deletion of this region results in a constitutively active kinase (Poteet-Smith et al., 1999).

The importance of RSK in homeostasis can be inferred by the observed defects in organ and tissue development and maintenance as a result of mutation or loss of RSK. For example, inactivating mutations in RSK2 have been found to be the cause of the X-linked dominant Coffin-Lowry syndrome in humans, which is partially phenocopied in mice (Rogers and Abidi, 1993; Trivier et al., 1996). In addition to regulating cognitive function the mutation of RSK2 has been implicated in behavioral disorders in humans (Hanauer and Young, 2002). RSK4 deletions have been identified in nonspecific X-linked retardation in humans but the evidence is not conclusive (Yntema et al., 1999). Knockout of RSK2 in mice decreases fertility in the female and also causes a lactation defect (Ludwik et al., 2020). RSK3 knockout in mice also results in decreased fertility due to an ovulation defect (Madogwe et al., 2021). RSK1 and RSK2 are additionally involved in immune cell function in mice (Lin et al., 2008; Zaru et al., 2015). RSK4 knockout mice have been reported but analysis of these animals has not been published. Although the ability to generate RSK4 knockout mice seems counterintuitive to the data suggesting that RSK4 inhibits receptor tyrosine kinase signaling in extraembryonic tissue (Myers et al., 2004). RSK also regulates differentiation in the mammary gland (Pasic et al., 2011), melanocytes (Kosnopfel et al., 2023) and osteoblasts (Yang et al., 2004). In summary, these results show that, although the RSK family regulates aspects of homeostasis based on the defects observed by knockout or mutation, targeting RSK in disease states is a reasonable strategy as RSK1/2/3 knockout mice are viable (Laugel-Haushalter et al., 2014).

## 2 RSK targets

### 2.1 Identifying targets

RSK functions in homeostasis and disease are regulated by direct substrate phosphorylation or by the interaction of RSK with specific binding partners (Gogl et al., 2018). The RSK NTKD preferentially phosphorylates a motif consisting of ArgXArgXXSer/Thr where X is any amino acid and Ser is the predominant phosphorylation site (Moritz et al., 2010). However, this motif is also preferentially phosphorylated by AKT and S6K (Fricke et al., 2023). In particular, identification of the physiologically relevant kinase responsible for substrate phosphorylation in cell-based assays or *in vivo* can be problematic as inhibitors, overexpression of wild type or dominant negative constructs and knockdown/knockout approaches have limitations. Regardless of the claims of specificity small molecule inhibitors invariably have off-target effects, which may vary in significance depending on the cell type and its environment. To offset these concerns the use of multiple small molecule inhibitors to the same target that have differing chemical structures and most likely differing off-target activities will provide confidence in validating target substrates. However, in addition to disparate *in vitro* inhibition constants the cell permeability and pharmacokinetic properties can be dissimilar between inhibitors and these factors need to be taken into account. Furthermore, these properties can be influenced by the cell/tissue type. Overexpression of wild type kinases provides the least convincing type of target validation as non-physiological expression levels will result in improper subcellular localization and altered kinase to substrate ratios, which potentially could result in promiscuous phosphorylation activity. Dominant negative kinases can also be problematic as they usually act by tightly binding to substrates but expression at non-physiological levels can result in interaction with non-specific targets. Knockdown approaches also have their difficulties as usually the kinase expression level is reduced but not absent and depending on the kinase and its activation state even a substantial reduction in the levels may be insufficient to reduce target phosphorylation. In contrast, small molecule inhibitors used at concentrations ten-fold above their IC_50_ will, in general, completely inhibit the kinase. These observations may account for the discrepancies observed between silencing *versus* small molecule approaches (Roffe et al., 2015). Knockout approaches provide a more definitive identification of physiological targets especially when combined with a rescue. However, hyperactivation of compensatory pathways or redundancy in target activation could confound the observations of the knockout. Additionally, in interpreting a knockout it should be considered whether the effects are due to loss of the catalytic or binding activity or both by the kinase. To aid in evaluating identified RSK substrates Supplementary Table S1 indicates the type of data used to support the claim.

**FIGURE 2:**
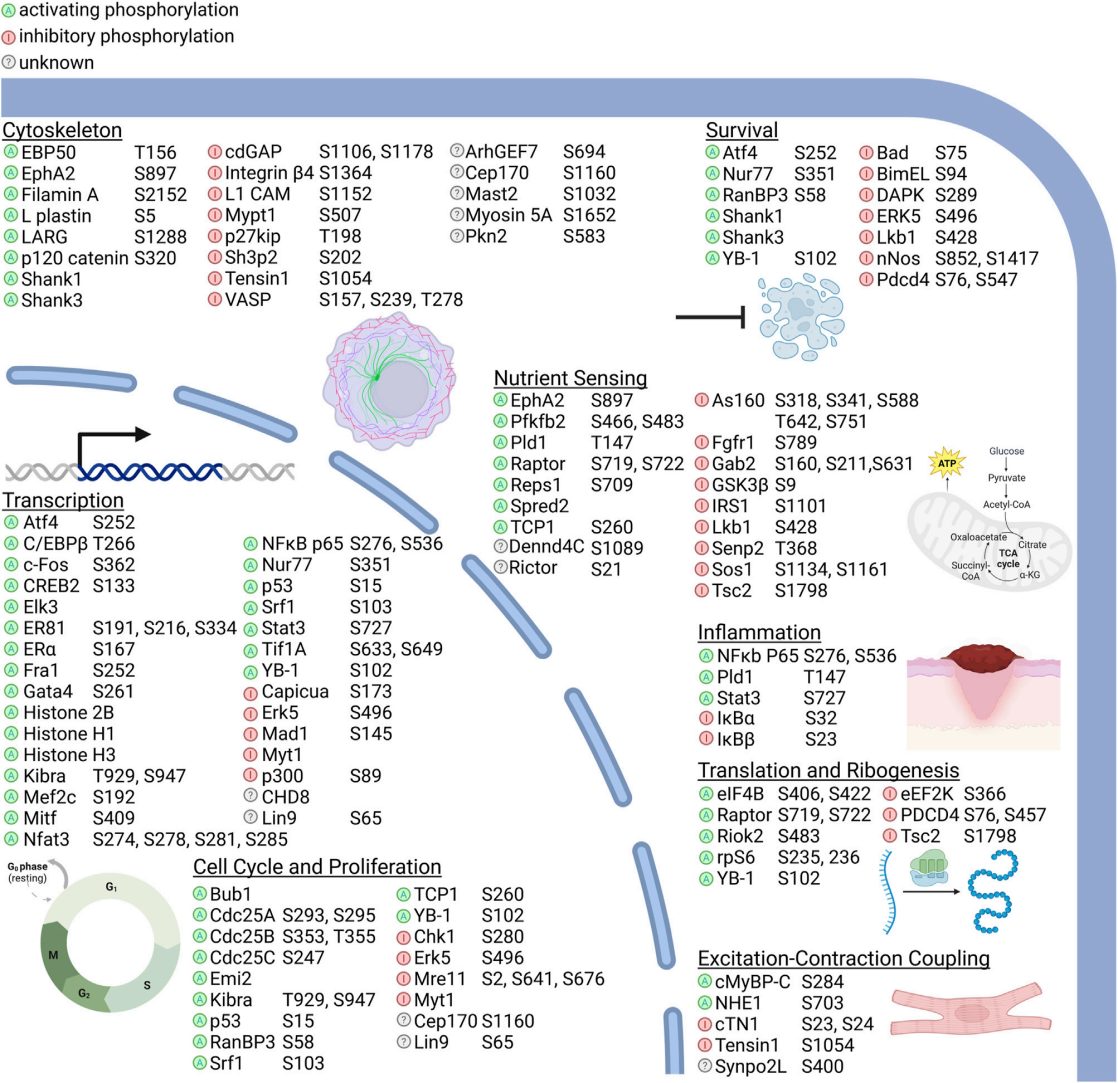
RSK substrates and their molecular functions. RSK kinase activity can activate or inhibit targets directly by the effect of phosphorylation, by regulating subcellular localization, or by introducing or inhibiting binding sites for other proteins. Substrates identified only by phosphoproteomic screens without subsequent description of the effects of RSK phosphorylation are listed as unknown. RSK substrate functions can be broadly classified into transcription, translation and ribosomal biogenesis, cell cycle regulation and proliferation, survival, nutrient sensing, excitation-contraction coupling, and inflammation. Nutrient sensing includes response to intracellular and extracellular metabolites and metals and ions. Phosphorylation sites are shown for each protein relative to amino acid numbering in the human protein, except for where the phospho site is unknown or the corresponding site is a non-phosphorylatable residue in human. A comprehensive list of RSK phosphorylation sites, motifs, and the validation methods used are detailed in Supplementary Table S1.

**FIGURE 3:**
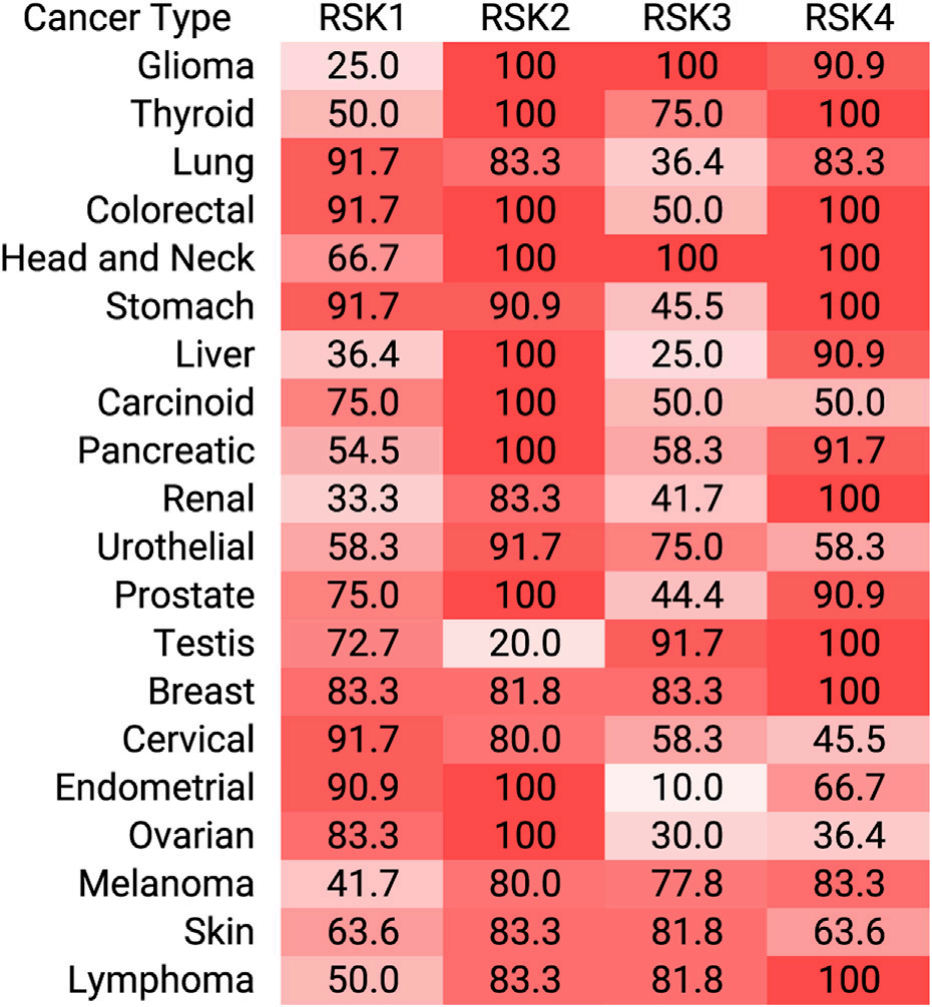
RSK isoform overexpression in cancer. Immunohistochemistry staining of tumors for each RSK isoform are assigned as high, medium, low relative to normal tissue. For each tumor and RSK isoform, the percent high or medium are shown. These data were obtained from the Human Protein Atlas.

RSK direct substrates can be broadly defined in the following categories: transcription, translation and ribosome biogenesis, proliferation and survival, cytoskeleton, nutrient sensing, excitation and inflammation (Figure 2). Target importance in a disease state is frequently evaluated based on whether the target is overexpressed in the effected organ or tissue compared to the non-disease state. Expression levels of the RSK isoforms has been most extensively evaluated in various cancers and an overview of the protein expression levels is presented (Figure 3). These data are based on relative expression in the disease state compared to non-disease using immunohistochemistry. A caveat of this data is that the total protein levels are not necessarily reflective of the amount of active kinase. Nonetheless, the data demonstrate that individual RSK isoforms are more highly expressed in some cancers than others, which would suggest that the overexpressed isoform may be involved in the tumorigenesis process. Additionally, the RSK isoforms vary in the cancer in which overexpression occurs and based on this observation it could be argued that the isoforms have non-overlapping functions.

An example of a well validated RSK target is estrogen receptor alpha (ERα), a known driver of estrogen receptor positive (ER+) breast cancer (Corti et al., 2023). Active RSK is correlated with responsiveness to endocrine-based therapies and overall survival (Jiang et al., 2007), and is present in the majority of locally advanced breast cancers (Moon et al., 2012). These results argue that a RSK inhibitor would be beneficial in the treatment of ER + breast cancer but because RSK regulates many cellular processes a RSK inhibitor is also likely to be effective in cancers that are ER negative (ER-). In ER-breast cancer the phosphorylation of Ser102 in YB-1, a transcription factor and regulator of mRNA translation is being used as a readout in a clinical trial with the RSK inhibitor, PMD-026 (Alkrekshi et al., 2021). However, as mentioned these substrates can also be phoshorylated by AKT or S6 and a decrease in phosphorylation will only be observed if RSK is the major driver of phosphorylation in the tissue or organ or disease state being analyzed.

### 2.2 RSK as a negative ERK1/2 regulator

**FIGURE 4:**
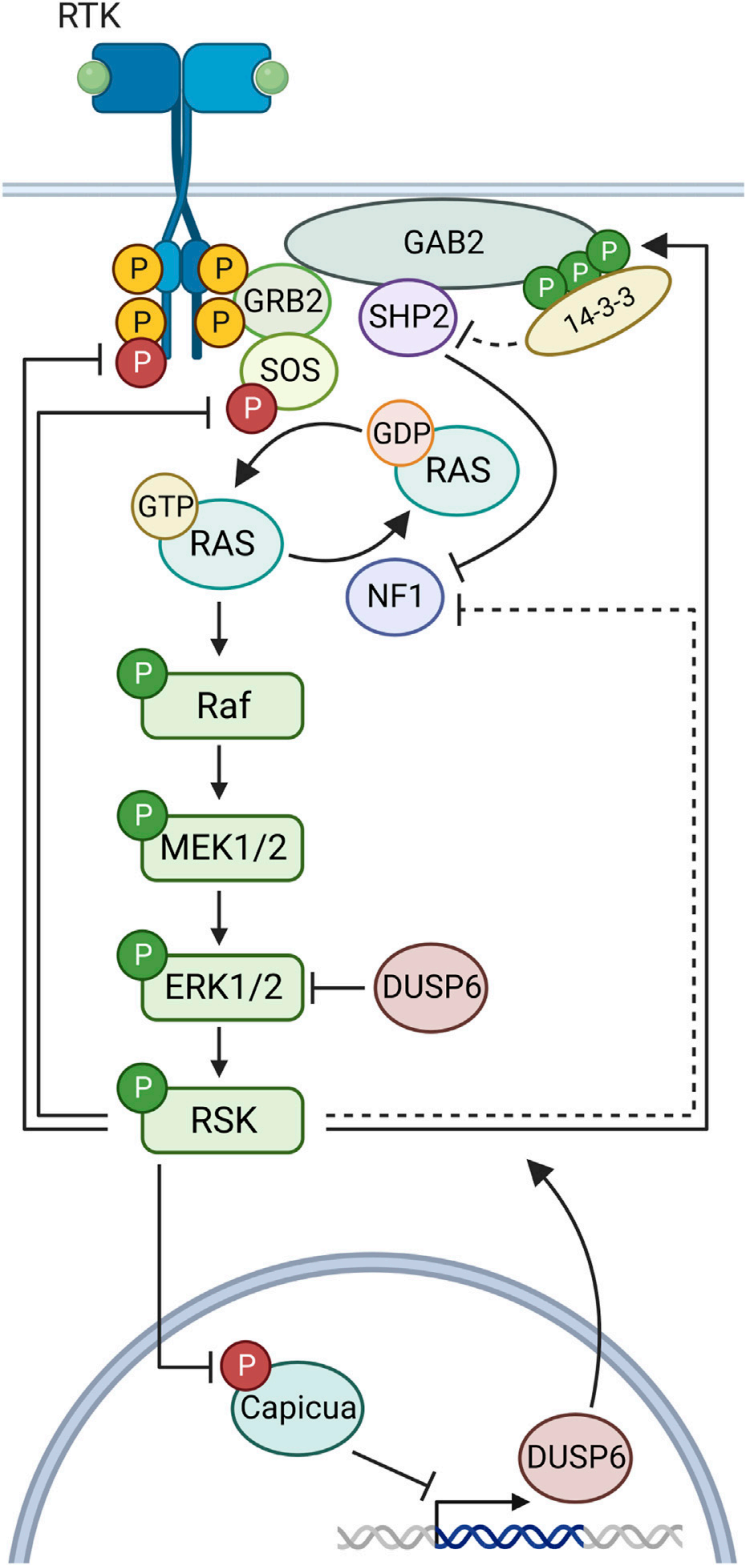
Negative feedback in the MAPK pathway by RSK. Ligand binding to a receptor tyrosine kinase (RTK) initiates docking of GRB2 and SOS, which then activates Ras to its GTP-bound form. Sequential phosphorylations lead to RSK activation. RSK phosphorylates sites on FGFR1, SOS1 to inhibit their function in the MAPK cascade. RSK multi-phosphorylation of GAB2 creates a docking site for 14-3-3 proteins, which block SHP2 complexing with Gab2. RSK inhibits the Ras guanine exchange factor NF1 through an unknown mechanism. RSK phosphorylates capicua to relieve transcriptional suppression of DUSP6.

The ability of RSK to act as a negative regulator of ERK1/2 activity substantially expands the number of physiological outcomes influenced by RSK as ERK1/2 is a global regulator governing numerous development (Rauen, 2013) and disease processes (Drosten and Barbacid, 2020). To prevent serious pathological consequences multiple mechanisms have evolved to downregulate the activity of the ERK1/2 pathway with the predominant mechanism probably varying according to cellular context (Figure 4). A negative feedback loop between RSK and ERK1/2 was first reported in *Drosophila*, in which RSK inhibits ERK1/2 nuclear localization and loss of RSK results in developmental abnormalities (Kim et al., 2006). In this mechanism the physical association between RSK and ERK1/2 is proposed to prevent ERK1/2 nuclear translocation and thereby, prevent ERK1/2 nuclear activity. Negative feedback is also important in mammalian development as RSK inhibition of ERK1/2 is necessary for fertility by maintaining estrogen responsiveness during the estrous cycle (Ludwik et al., 2020) and also maintenance of naïve pluripotency (Nett et al., 2018). Negative inhibition of ERK1/2 in these mammalian systems occurs through unidentiifed RSK kinase dependent mechanisms. Thus RSK is able to inhibit ERK1/2 signaling through physical association and substrate phosphorylation depending on the cellular context.

Analysis of the regulatory pathways by which RSK inhibits ERK1/2 has identified a number of different mechanisms (Figure 4). Upstream of ERK1/2, RSK2 interacts and phosphorylates fibroblast growth factor receptor 1 (FGFR1) (Figure 2 and Supplementary Table S1) to reduce FGFR1 downstream signaling (Nadratowska-Wesolowska et al., 2014). This reduction in pathway activation results in decreased ERK1/2 activity. RSK can also inhibit MAPK pathway activation by decreasing RAS activation (Zhang et al., 2013). In this mechanism RSK phosphorylation of the adapter protein, GAB2, reduces the ability of the tyrosine phosphatase, SHP2, to interact with GAB2. Inhibition of SHP2 binding decreases RAS activation by multiple mechanisms (Neel et al., 2003; Hanafusa et al., 2004; Bunda et al., 2015). Interestingly, RSK2 phosphorylation of GAB2 does not alter phosphoinositide 3-kinase activation. Moreover, RSK can reduce RAS activation by phosphorylating the RAS GTPase exchange protein, SOS1 (Douville and Downward, 1997; Saha et al., 2012) (Figure 2 and Supplementary Table S1). This SOS1 phosphorylation is hypothesized to result in the interaction with an adaptor 14-3-3 protein to inhibit RAS interaction. Furthermore, RSK has also been shown to phosphorylate the Ras GTP-hydroylase activating protein, neurofibromin, to promote GTP hydrolysis and thereby, reduce Ras activation (Hennig et al., 2016).

Downstream of ERK1/2, RSK negatively regulates ERK1/2 activation by promoting the transcription of dual specificity phosphatase 6 (DUSP6) (Ren et al., 2020). DUSP6 is a member of the large DUSP family that comprises phosphatases that dephosphorylate and inactivate the various MAPKs, and DUSP6 is specific to ERK1/2 (Chen et al., 2019). RSK promotes DUSP6 expression by phosphorylation of the transcriptional repressor Capicua (Figure 2 and Supplementary Table S1). This phosphorylation results in the interaction of an adaptor 14-3-3, which promotes the nuclear export of Capicua to relieve the repression of DUSP6 expression (Dissanayake et al., 2011; Galan et al., 2014; Ren et al., 2020). In summary, the multitude of negative regulatory mechanisms by which RSK regulates ERK1/2 clearly demonstrates the importance of RSK in ERK1/2-mediated physiological processes.

## 3 RSK inhibitors

### 3.1 Introduction

**FIGURE 5:**
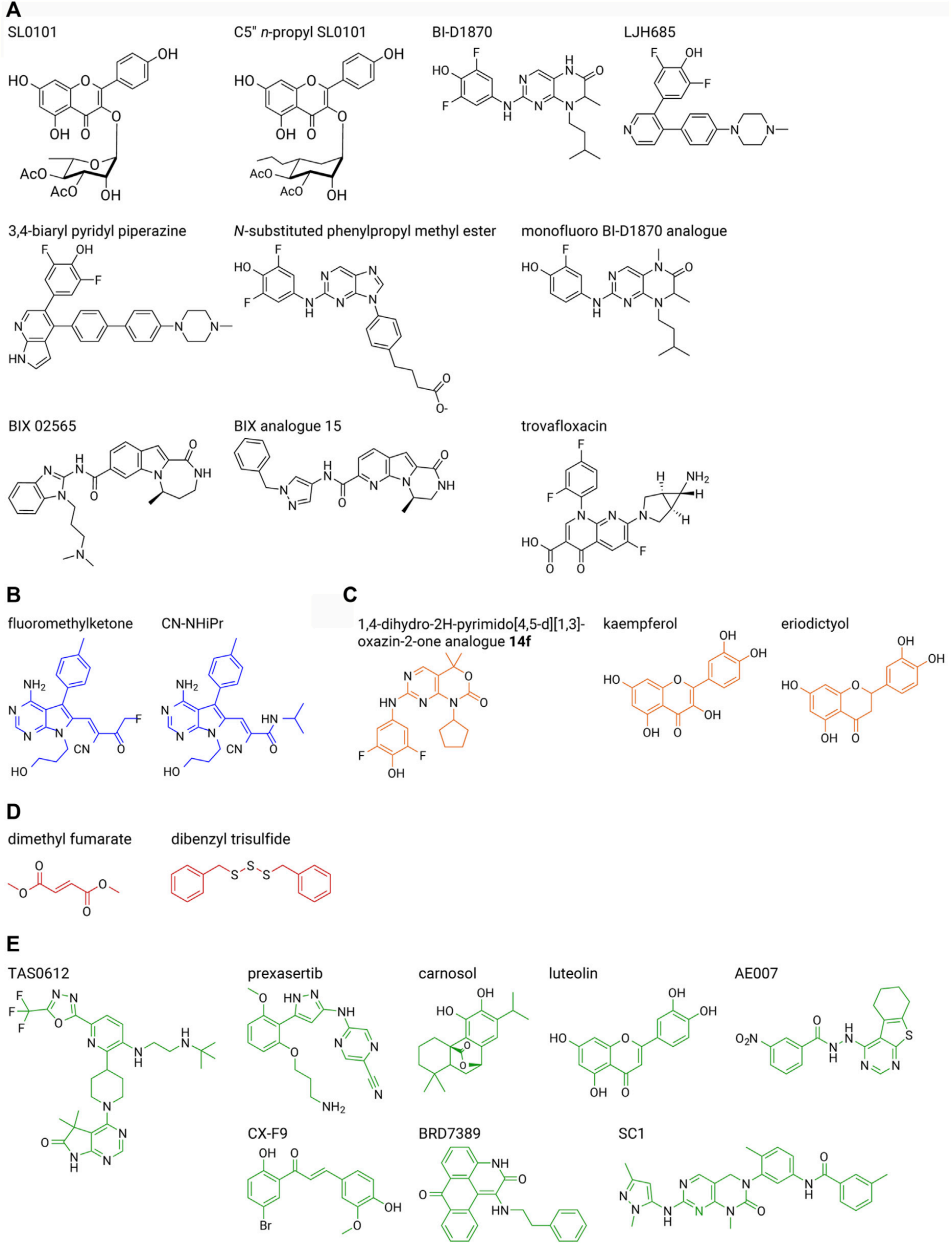
RSK inhibitor structures. Select inhibitors are shown from each series described in the text. **(A)** RSK-specific NTKD inhibitors (black), **(B)** RSK-specific CTKD inhibitors (blue), **(C)** non-specific NTKD inhibitors (orange), **(D)** non-specific CTKD inhibitors (red), **(E)** other non-specific inhibitors (green). A list of inhibitory efficacy of the various compounds in vitro kinase and cell-based assays is provided in Supplementary Tables S2, S3.

RSK inhibitors, PMD-026 (Ushijima et al., 2022), and TAS0612 (Shibata et al., 2020), discussed further below, are in early stage clinical trials, NCT04115306 and NCT04586270, respectively, for metastatic solid tumors. In this section we will also discuss these agents as well as specific and non-specific inhibitors of the NTKD or CTKD, which are being evaluated in the pre-clinical stage of drug development. Interestingly, small molecule inhibitors for RSK have not been as extensively developed as for other components of the MAPK family, e.g., there are numerous inhibitors that are FDA-approved or in clinical trials for mutant KRAS, mutant BRAF, MEK1/2 and ERK1/2 (Song et al., 2023). In part, this lack of interest in developing RSK inhibitors was based on the opinion that targeting global regulators of the ERK1/2 pathway would be more effective at disease prevention than targeting just the subset of RSK-regulated targets. However, inhibition of a global regulator can result in increased side effects as demonstrated by the observation that in some cancers MEK1/2 inhibition activates the PI3K/AKT pathway (Mendoza et al., 2011). In contrast RSK inhibition by the small molecule analogues of SL0101 do not lead to PI3K/AKT activation (Ludwik et al., 2016). Supplementary Tables S2, S3 provide a summary of the inhibitory potency of the various compounds *in vitro* kinase and cell-based assays including analogues that are discussed in this Section (Figure 5).

Small molecule kinase inhibitors for Ser/Thr kinases are classified into different types based partly on the position of a highly conserved Asp-Phe-Gly (DFG) motif, which has previously been reviewed (Lee et al., 2023). In common with other Ser/Thr protein kinases the tertiary structures of the NTKD and CTKD are comprised of a small N-terminal lobe and a larger C-terminal lobe. The activation loop is present within the C-lobe and contains the DFG motif [[expanded view Figure 6A (active NTKD) and 6E (inactive CTKD)]. In the active kinase conformation the DFG motif faces into the interior of the kinase or “in” and interacts with the ATP phosphates. In the inactive kinase conformation or “out” the DFG motif faces away from the ATP-binding site. Based on available crystal structure information the small molecule reversible inhibitors for the RSK NTKD and CTKD are type II inhibitors in which the inhibitor is bound to a DFG “out” conformation. The irreversible CTKD inhibitors discussed are classed as type I based on their binding in the ATP pocket and “DFG” in conformation. In some of the cases discussed the ability of the small molecule to interact with the isolated NTKD or CTKD was not determined and therefore, these compounds are generically referred to as RSK inhibitors.

**FIGURE 6:**
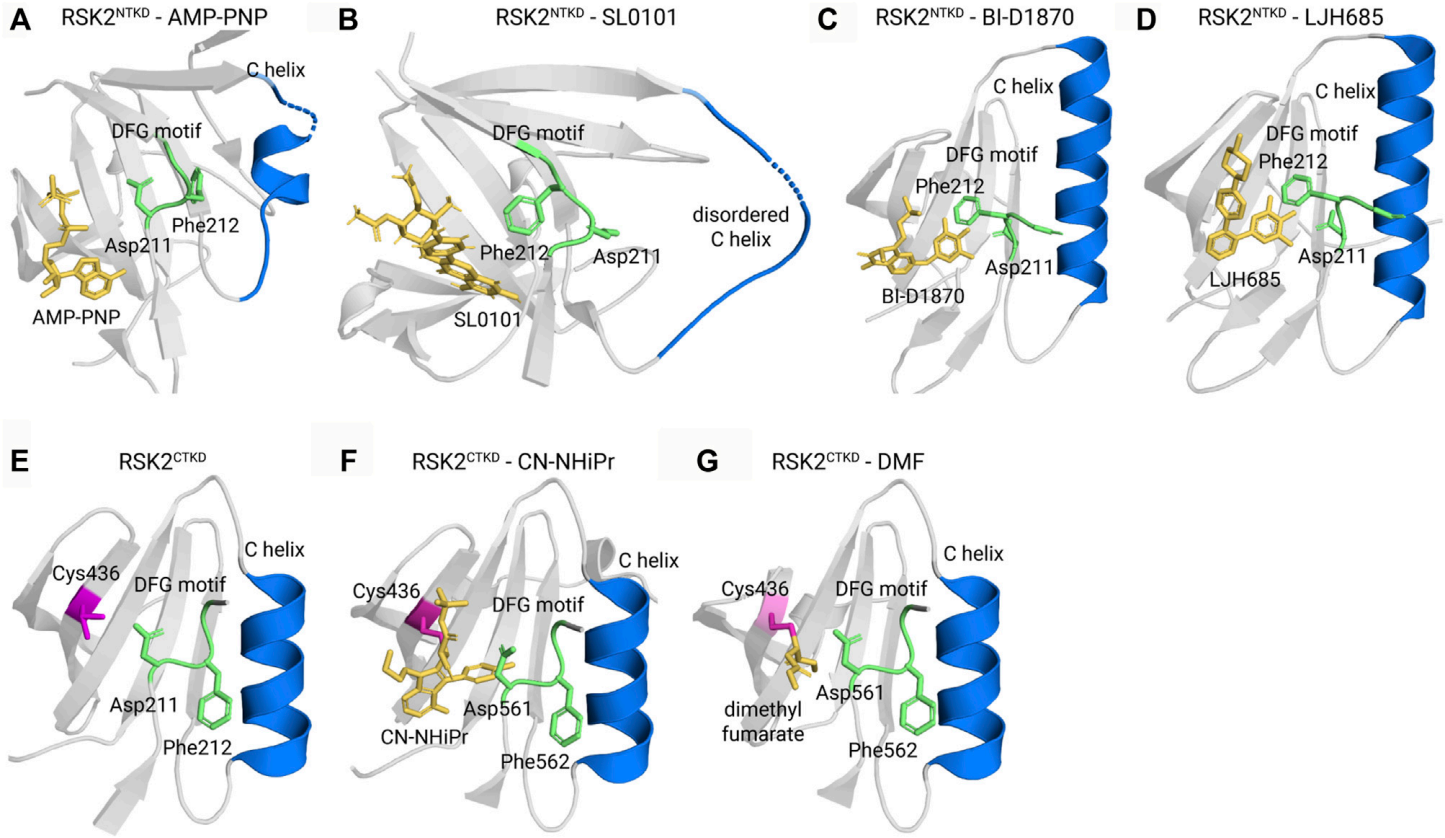
Kinase-inhibitor crystal structures. **(A)** The active kinase in complex with the ATP surrogate AMP-PNP (Protein Data Bank ID 3G51) adopts a DGF motif-in, α C helix-in conformation. In Type II inhibitors, such as **(B)** SL0101 (PDB ID 3UBD), **(C)** BI-D1870 (5D9K), and **(D)** LJH685 (PDB ID 4NUS), the DFG motif flips out to face away from the ATP binding pocket. **(E)** The unliganded CTKD (PDB ID 2QR8) adopts an inactive conformation. **(F)** The covalent inhibitors CN-NHiPr (PDB ID 4D9U) and **(G)** dimethyl fumarate (PDB ID 5O1S) are Type I inhibitors that adopt a DFG-in, C-in conformation and covalently bond to a reactive cysteine, C436.

### 3.2 NTKD inhibitors

#### 3.2.1 SL0101 series

The first specific inhibitor of RSK, SL0101, was isolated from an extract obtained from *Forsteronia refracta* in a screen of botanical extracts (Smith et al., 2005) (Figure 5A and Supplementary Tables S2, S3). SL0101 is not a pan-RSK inhibitor as has been mistakenly referred to in literature reports. SL0101 specifically inhibits RSK1 and RSK2 NTKD activity with no effect on RSK3 and RSK4 NTKD activity (Wright et al., 2021). SL0101 has an unusual binding mechanism in which the NTKD undergoes a structural rearrangement that creates a hydrophobic pocket to accommodate SL0101, and as a result disrupts the ATP-binding pocket (Utepbergenov et al., 2012) (compare Figures 6A,B). Computational modeling of the NTKDs of the other RSK family members are consistent with the ability of the RSK1 and RSK2 NTKDs to form the SL0101 binding pocket whereas this pocket is sterically inhibited from forming in the RSK3 and RSK4 NTKDs (Wright et al., 2021). In a screen of 71 kinases, SL0101 (10 μM) inhibited RSK1 and RSK2 but also inhibited Aurora B and PIM3 (Bain et al., 2007). Unfortunately, the use of SL0101 is limited for *in vitro* use and it is rapidly metabolized (Mrozowski et al., 2012). However, there is a report that intraventricular infusion of SL0101 into the brain resulted in alterations of the circadian clock (Hoyt et al., 2023). Drug metabolism differs within and outside the central nervous system (CNS) compartment (Cole et al., 2015) and it is unknown whether SL0101 has a more suitable pharmacokinetic profile in the CNS, which may account for the effects on the circadian clock. SL0101 reduced viral replication in a vaginal model of Herpes simplex virus type I infection but the delivery route for SL0101 was not described (Ding et al., 2019).

Structure-activity-relationship (SAR) studies using SL0101 as the lead compound were used to identify the analogue C5″ *n*-propyl SL0101 (Figure 5A) (Smith et al., 2006; Smith et al., 2007; Hilinski et al., 2012; Mrozowski et al., 2012; Mrozowski et al., 2014; Li et al., 2015; Ludwik et al., 2016; Li et al., 2017; Li et al. 2020a; Li et al. 2020b; Wright et al., 2021) (Supplementary Tables S2, S3). This analogue has an improved potency of two-fold in RSK2 *in vitro* kinase assays (IC_50_–392 nM vs. 183 nM (10 μM ATP)) and five-fold in cell-based assays compared to SL0101 (IC_50_ 50 μM vs. 8 μM in an MCF-7 cell line). In a screen of 247 kinases C5″ *n*-propyl SL0101, RSK1 and RSK2 were the top hits, and the next most significantly inhibited targets were CSF1R and MAP4K4 (Ludwik et al., 2016). In a rigorous test of target specificity in cell-based assays C5″ *n*-propyl SL0101 was unable to further inhibit proliferation of the TNBC cell line, MDA-MB-231, when RSK1 and RSK2 were knocked down (Ludwik et al., 2016). Analysis of the kinetics of C5″ *n*-propyl SL0101 showed that its off rate is much lower than that of SL0101 (Wright et al., 2021). Therefore, C5″ *n*-propyl SL0101, theoretically, should have an improved pharmacokinetic profile compared to SL0101 (Mrozowski et al., 2012). A slow off rate would make a complex of RSK2 with C5″ *n*-propyl SL0101 more stable than with SL0101, resulting in prolonged inhibition of RSK2 kinase activity and protection of C5″ *n*-propyl SL0101 against metabolism *in vivo*. This hypothesis is supported by the observations that C5″ *n*-propyl SL0101 showed sustained inhibition of MCF-7 cell proliferation *in vitro* (>96 h) compared to SL0101 (<48 h) (Ludwik et al., 2016). Additionally, *in vivo*, C5″ *n*-propyl SL0101 altered phosphorylation of S6 and eukaryotic elongation factor 2, which are known RSK downstream effectors (Ludwik et al., 2016; Ludwik et al., 2018) (Figure 2). Consistent with these on-target effects C5” *n*-propyl SL0101 showed anti-cancer efficacy *in vivo* as TNBC metastatic tumor burden was reduced in a xenograft model (Ludwik et al., 2016). Importantly, analogues based on SL0101 do not result in activation of AKT as has been observed with MEK1/2 inhibitors (Mendoza et al., 2011). These observations support the further development of the SL0101 series for *in vivo* use.

#### 3.2.2 BI-D1870 series

BI-D1870 was identified in a screen for polo-like kinase 1 inhibitors (PLK1) and was found to have an IC_50_ for PLK1 of ∼100 nM ([ATP] 50 µM). However, BI-D1870 was also found to be an ATP-competitive pan-RSK NTKD inhibitor with IC_50_s ranging from −15 to 31 nM ([ATP] 100 µM) (Sapkota et al., 2007) (Figures 5A, 6C). In subsequent specificity analysis BI-D1870 substantially inhibited Aurora B, MELK and MST2 (Bain et al., 2007). In a screen for inhibitors of JAK2 pseudokinase domain, BI-D1870 was also identified as a JAK2 inhibitor with an IC_50_ of −654 nM (McNally et al., 2019). Further off target effects by BI-D1870 were identified using proteomic approaches in dendritic cells, which highlighted concerns about interpreting the importance of RSK in dendritic cells (Edgar et al., 2014). BI-D1870 was also found to interact with bromodomain-containing protein 4 (BRD4) with a K_d_ of −3.5 µM by isothermal titration calorimetry (Ciceri et al., 2014) but an equivalent measure of BI-D1870 for RSK has not been reported. Measuring inhibition of cellular RSK2 in a proximity-based assay gave an IC_50_ of −10 µM in a human acute myeloid leukemia cell line, MOLM-13, (Casalvieri et al., 2020). However, in subsequent reports using the proximity-based assay the IC_50_ for BI-D1870 in MOLM-13 was reported to be <200 nM, raising concerns about the reproducibility of the assay (Casalvieri et al., 2021). In many cell-based assays BI-D1870 is used at concentrations of −10 μM (Roffe et al., 2015) (Supplementary Tables S2, S3). BI-D1870 was found to be challenging to formulate for *in vivo* administration and was also found to have a poor pharmacokinetic profile (Pambid et al., 2014). There are reports of *in vivo* efficacy of BI-D1870 to reduce the symptoms of experimental autoimmune encephalomyelitis using 0.5 mg/kg but whether these effects were due to on-target RSK inhibition were not identified (Takada et al., 2016). Subcutaneous neuroblastoma tumor growth was also reduced using 10–40 mg/kg but on target RSK inhibition was not reported and the authors proposed that BI-D1870 was inhibiting the PI3K-AKT-MTORC1 pathway but the mechanism for this inhibition was not presented (Jin et al., 2023).

In a screening effort to identify BI-D1870 derivatives with improved *in vivo* properties a bis-phenolpyrazole scaffold was identified in a high throughput screen and from this lead compound a 3,4-biaryl series was synthesized and evaluated (Jain et al., 2015) (Supplementary Table S2). The best inhibitor in this series, LJH685, considered a pseudo-analogue of BI-D1870, is also a pan-RSK inhibitor but has a higher affinity and specificity for RSK than BI-D1870 (Aronchik et al., 2014) (Figures 5A, 6D and Supplementary Table S2). However, LJH685 does not demonstrate improved efficacy in cell-based assays compared to BI-D1870 and metabolic liabilities remain (Jain et al., 2015; Casalvieri et al., 2020) (Supplementary Table S3). In an attempt to improve the pharmacokinetic properties of LJH685 a series of bi-aryl pyridyl analogues were generated but in cell-based assays the best analogue had an efficacy similar to LJH685 although the concentrations of inhibitors used in these assays was not identified (Cui et al., 2022) (Figure 5A and Supplementary Table S2). In an additional attempt to improve the efficacy of BI-D1870 the 7-azaindole series was developed from a 3,4-bi-aryl pyridyl series and an analogue with picomolar affinity was identified with selectivity as a pan RSK inhibitor but pharmacokinetic issues remained (Jain et al., 2018) (Supplementary Table S2). A more extensive structure-activity relationship study of BI-D1870 incorporated substitutions in the pteridinone and pyrimidine rings with no modification substantially improving the IC_50_ based on *in vitro* RSK2 kinase or cell-based assays with MOLM-13 cells (Casalvieri et al., 2020) (Supplementary Table S2). BI-D1870 was further modified by introducing N-substituted pyrrolpyrimidine and purine rings but substantial improvements in IC_50_s obtained from RSK2 *in vitro* kinase or cell-based assays were not obtained (Casalvieri et al., 2021) (Supplementary Tables S2, S3). An analogue with an N-substitution of a phenylpropyl methyl ester showed similar efficacy as BI-D1870 at inhibiting RSK2 *in vitro* and in a cell-based proximity assay but showed five-fold reduced potency with cell-based assays using MOLM-13 cells (Figure 5A and Supplementary Tables S2, S3). The authors argue from these data that RSK inhibition can be separated from the cytotoxic effects of these compounds on MOLM-13 cells. Consistent with this argument Casalvieri et al. identified a monofluoro BI-D1870 analogue that had similar efficacy to BI-D1870 *in vitro* for RSK2 and in cell-based assays using MOLM-13 cells but did not inhibit RSK2 in a cell-based proximity assay (Casalvieri et al., 2020). However, the ability of the compounds to inhibit RSK2 in the cell-based proximity assay was performed in HEK293 cells and whether RSK inhibition occurred in MOLM-13 cells was not evaluated. Therefore, the effects in MOLM-13 cells could be due to off target effects of the analogues (Chae et al., 2020). In general, despite these synthetic efforts, an improvement in the drug-like properties for BI-D1870 has not succeeded.

#### 3.2.3 BIX 02565

The 1-oxo-2,3,4,5-tetrahydro-1H-[1,4]diazepinol [1,2-a]indole-8-carboxamide scaffold was used as the basis to identify novel inhibitors of RSK (Boyer et al., 2012). In subsequent SAR of this indole series the inhibitor, BIX 02565, was identified and had an IC_50_ of 1 nM for RSK2 in an *in vitro* kinase assay (Figure 5A and Supplementary Table S2). However, this compound also inhibited other kinases in the nM range (Kirrane et al., 2012). Moreover, in subsequent analysis BIX 02565 was identified to inhibit several adrenergic receptor subtypes and the imidazoline I_2_ receptor (Fryer et al., 2012). *In vivo* BIX 02565 was found to inhibit NHE1 activity and improve cardiac function in an ischemia/reperfusion model (Shi et al., 2015). However, it is not clear whether these effects were due to inhibition of RSK because of the reported off target effects of BIX 02565. Additional SAR studies of BIX 02565 led to a compound with an *in vitro* IC_50_ of 0.2 nM and in cell-based assays of 0.32 nM (Fryer et al., 2012) (Figure 5A). However, no further reports on this BIX 02565 analogue appear in the literature.

#### 3.2.4 Quinolone antibiotics

Several derivatives of the floxacin antibiotics were identified as RSK4 inhibitors and of these trovafloxacin was the most fully characterized and is a non-competitive ATP inhibitor with an *in vitro* IC_50_ of −2.5 μM for RSK4 (Chrysostomou et al., 2021) (Figure 5A and Supplementary Table S2). Trovafloxacin was selective for RSK4 in an *in vitro* screen of 140 kinases and silencing of RSK4 reduced the ability of trovafloxacin to enhance cisplatin-induced apoptosis in the lung cancer line, A549. Indirect evidence is consistent with trovafloxacin binding to an allosteric site in the RSK4 NTKD. *In vivo* trovafloxacin administration by oral gavage reduced tumor growth but whether this effect was an on target action of the drug was not evaluated. Trovafloxacin has an excellent pharmacokinetic profile but due to the high risk of hepatotoxicity is reserved for life- or limb-threatening infections (Moellering, 1998).

#### 3.2.5 PMD-026

The compound PMD-026 is the first RSK inhibitor in clinical trials and a multicenter phase 1/1b trial in the United States in patients with metastatic and triple negative breast cancer is ongoing (NCT04115306). In meeting abstracts PMD-026 has been described as orally bioavailable with a good safety profile in pre-clinical models (Dunn et al., 2018). The clinical study overview states that PMD-026 is a pan RSK inhibitor with high selectivity for RSK2. The structure of the compound is not provided although the sponsor of the trial has a patent for substituted tetrahydropyrido [3′,2′:4,5]pyrrolo [1,2-α]pyrazine-2-carboxamides as p90 RSK inhibitors (Supplementary Tables S2, S3). It is unknown whether PMD-026 belongs to this class of inhibitors. PMD-026 decreased proliferation in a prostate cancer line, 22 Rv1, expressing an androgen receptor variant (Ushijima et al., 2022). *In vivo* PDM-026 inhibited the growth of 22Rv1 tumors and sensitized the tumors to the androgen antagonist enzalutamide. The on target action of PMD-026 was not reported in these xenograft studies. PMD-026 has also been shown to inhibit the proliferation *in vitro* and *in vivo* of melanoma lines with MAPK hyperactivation (Kosnopfel et al., 2023). The phosphorylation of YB-1 was used as a biomarker to show on target inhibition of RSK by PMD-026 (Figure 2 and Supplementary Table S1). Interestingly, RSK inhibition by PMD-026 or BI-D1870 increased expression on the major histocompatibility complex (MHC) class 1 (MHC-1), and downregulation of MHC-1 is associated with tumor immunity (Balasubramanian et al., 2022). These observations are the first to demonstrate a link between RSK and tumor immunology. As PMD-026 is a pan-RSK inhibitor the identity of the RSK isoform responsible for its various biological actions has not been identified.

### 3.3 CTKD inhibitors

#### 3.3.1 Fluoromethylketone

Cohen, et al., using a bioinformatics approach, identified that the CTKD of RSK1, RSK2, and RSK4 have a threonine in the gatekeeper region and a reactive cysteine within the ATP-binding site residue which could form a covalent bond with an inhibitor in the ATP pocket (Cohen et al., 2005). Electrophilic pyrrolopyrimidines were selected as lead compounds and fluoromethylketone (termed FMK) was identified as a covalent RSK2 CTKD inhibitor (Figure 5B). RSK3 has a methionine in the gatekeeper region that by steric hindrance prevents RSK3 inhibition by FMK. FMK is an irreversible CTKD inhibitor with an IC_50_ of −15 nM [100 μM ATP] in an *in vitro* kinase assay (Supplementary Table S2). The only known function of the RSK CTKD is autophosphorylation, which contributes to the activation of the NTKD. In an *in vitro* kinase assay significant inhibition of LCK, CSK and p70S6 kinase 1 were observed at concentrations of FMK that did not inhibit RSK1 and RSK2 (Bain et al., 2007). In a cell-based assay FMK inhibited the PMA-induced CTKD autophosphorylation of Ser-386 with an IC_50_ of −150 nM. However, once active, a CTKD inhibitor cannot inhibit RSK activity, and in some contexts the CTKD is not required for NTKD activation, thereby limiting FMK utility (Cohen et al., 2007; Zaru et al., 2007). However, it is possible to improve the usefulness of a CTKD inhibitor by using it to target degradation of RSK1, RSK2 and RSK4 through the ubiquitin-proteasome pathway. In this approach a heterobifunctional small molecule is generated that is capable of interacting with the target such as FMK and also separately able to recruit an E3 ubiquitin ligase. These heterobifunctional small molecules are referred to as proteolysis-targeting chimeras (PROTAC) and several are in clinical trials (Bekes et al., 2022).

#### 3.3.2 CN-NHiPR

Covalent inhibitors are attractive because they are often specific and have improved pharmacological properties over reversible inhibitors (Ghosh et al., 2019). Aspirin and penicillin are classical examples; however, covalent drug metabolites have shown toxicity and drug-protein adducts can be immunogenic. Therefore, Serafimova et al. designed and identified inhibitors that also targeted the cysteine thiols in the RSK1, RSK2, and RSK4 CTKDs but which were reversible (Serafimova et al., 2012). Of these the N-isopropyl cyanoacrylamide, CN-NHiPr, was selected for additional analysis and demonstrated a sub-nanomolar affinity for the RSK1 CTKD to inhibit autophosphorylation in cell-based assays at < 10 nM [100 μM ATP] (Figures 5B, 6F). The use of this inhibitor beyond this initial report has not been presented in the literature.

### 3.4 Non-specific NTKD inhibitors

In a small screen for inhibitors of RSK4 NTKD compounds that contained a 1,4-dihydro-*2H*-pyrimido [4,5-*d*][1,3] oxazin-2-one demonstrated inhibition (Yuan et al., 2021). SAR studies identified compound 14f, which had an IC_50_ of −10 nM [10 μM ATP] in an *in vitro* kinase activity (Figure 5C). However, 14f inhibited other kinases >35% in the AGC and CAMK family at 100 nM. In cell based assays using esophageal squamous cell carcinoma (ESCC) the IC_50_ for inhibition of proliferation was −0.6 μM (Supplementary Tables S2, S3). *In vivo* metabolism was rapid (<1 h) but intraperitoneal daily injections of 50 mg/kg were found to reduce ESCC tumor growth. On target *in vivo* inhibition was validated by examining RPS6 and GSK3β phosphorylation.

Kaempferol is a naturally occuring flavonoid found in a wide variety of plants, which has been proposed to be benefical in a number of disease processes (Ren et al., 2019) (Figure 5C and Supplementary Tables S2, S3). The molecular targets of kaempferol have not been extensively identified although it has been proposed to be a RSK2 inhibitor (Cho et al., 2007). However, kaempferol inhibits a number of other kinases (Yao et al., 2014). In an *in vitro* kinase assay kaempferol was found to have an IC_50_ of −15 μM [10 μM ATP] (Smith et al., 2007) and therefore, its use as a chemical probe in cell-based assays would be limited. Eriodictyol, a member of the flavonoid family (Islam et al., 2020), was also proposed to be a RSK2 inhibitor (Liu et al., 2011) but its specficity for RSK2 has not been examined (Figure 5C).

### 3.5 Non-specific CTKD inhibitors

Dimethyl fumarate (DMF) is approved for treatment of multiple sclerosis and psoriasis because of antioxidative and anti-inflammatory properties (Bennett Saidu et al., 2018) (Figures 5D, 6G). DMF is in a number of clinical trials for various diseases. DMF inhibits the RSK2 CTKD in an *in vitro* kinase assay with an IC_50_ of −225 μM [200 μM ATP] through a mechanism in which DMF acts as a Michael acceptor to succinylate a critical cysteine residue. This succinylation results in autoinhibition of the CTKD by sterically inhibiting the movement of the activation loop (Andersen et al., 2018). In cell-based assays DMF also inhibited mitogen-and stress-activated kinase (MSK) and RSK1 (Gesser et al., 2011). However, in an *in vitro* kinase screen, DMF at 1 mM was unable to inhibit activated RSK1, RSK2 and MSK1 but inhibited PRK2 and PKCα (Andersen et al., 2018). Thus similar to other CTKD inhibitors DMF does not inhibit RSK once it is active. The efficacy of DMF to inhibit RSK2 CTKD increases over a 48 h window (Andersen et al., 2018). These data are consistent with the observations that DMF is only effective after it depletes glutathione by covalent modification (Campione et al., 2022). DMF physiological effects could be due to a number of mechanisms other than RSK/MSK inhibition. However, the efficacy and time course of RSK inhibition is consistent with clinical observations in which high doses and a prolonged time period are required for effective treatment of psoriasis.

Dibenzyl trisulfide isolated from the Jamaican Guinea Hen Weed was found to be relatively specific for inhibition of the RSK1 CTKD *in vitro* kinase assays with a K_d_ of −1.3 μM (Lowe et al., 2014) (Figure 5D). In cell based assay the IC_50_ for inhibition of proliferation of a number of cancer lines was <1 μM, which suggests that mechanisms in addition to RSK1 CTKD inhibition are important for its efficacy as the *in vitro* K_d_ is higher than the cell based IC_50_ (Lowe et al., 2014; Wooten et al., 2022). Dibenzyl trisulfide also inhibits cytochrome P450 proteins CYP1A1 and CYP1B1 with IC_50_s similar to that of RSK1 CTKD inhibition (Wauchope et al., 2021).

### 3.6 Non-specific RSK inhibitors

TAS0612 is a 5 h-pyrrolo [2,3-D]pyrimidin-6 (7H)-one derivative, which is a pan AKT inhibitor that also inhibits RSK and S6 kinase (Figure 5E and Supplementary Table S2) (Ichikawa et al., 2023). This multikinase inhibitor is in Phase 1 clinical trial for locally advanced or metastatic solid tumors (NCT04586270). TAS0612 inhibits proliferation of various breast cancer cell lines with IC_50_s ranging from −25 to 320 nM (Shibata et al., 2020) (Supplementary Table S3).

LY2606368 (prexasertib) is an ATP-competitive inhibitor of Chk1, Chk2 and RSK1 *in vitro* kinase assays with IC_50_s < 10 nM (King et al., 2015) (Figure 5E and Supplementary Table S2). LY2606368 inhibited CHK2 activation with an IC_50_ of −30 nM but did not inhibit RSK activation at a dose of 100 nM in cell-based assays. A number of clinical trials evaluating prexasertib in various cancers have been completed or are ongoing. In early 2023 the FDA granted a fast track designation to prexasertib for ovarian and endometrial cancers.

Carnosol is a natural product isolated from rosemary, sage and oregano and was reported to be a RSK2 inhibitor (Wang et al., 2018) (Figure 5E). In a small screen carnosol demonstrated selectivity for RSK2 but the IC_50_ in an *in vitro* kinase assay was −10 μm, raising concerns about its usefulness in cell-based assays (Supplementary Tables S2, S3). The selectivity for RSK2 has not been reported.

Luteolin, is a phytochemical with the formula 3,4,5,7-tetrahydroxy flavone and has been shown to have efficacy in a variety of *in vitro* and *in vivo* cancer models (Imran et al., 2019) (Figure 5E and Supplementary Table S3). Luteolin was reported to inhibit RSK1 with a IC_50_ −4 μM (Reipas et al., 2013) and RSK2 (Lim et al., 2013) but the specificity of luteolin has not been thoroughly examined (Supplementary Table S2).

Using computational approaches AE007 was identified as a potential RSK2 inhibitor (Zhao et al., 2021) (Figure 5E). RSK2 has a K_d_ of 856 nM for AE007 and in a pull down assays RSK2 was shown to interact with AE007 (Supplementary Table S2) (Zhao et al., 2021; Li et al., 2022). However, the specificity of AE007 for RSK2 was not evaluated and it is unknown whether the decreased *in vitro* proliferation and *in vivo* tumor growth in melanoma models was due to RSK2 inhibition (Supplementary Table S3).

The compound CX-F9 is also reported to be a RSK2 inhibitor and was also discovered by computational methods by the same group that identified AE007 (Zhang et al., 2019) (Figure 5E). CX-F9 also pulls down RSK2 but the K_d_ and specificity of CX-F9 was not reported (Supplementary Table S2)

BRD7389 was identified in a computational approach to identify compounds that induced insulin expression in pancreatic α cell (Fomina-Yadlin et al., 2010) (Figure 5E). BRD73879 appears to be a pan RSK inhibitor with IC_50_s ranging from −1 to 2 μM *in vitro* kinase assays but also inhibits numerous other targets (Supplementary Table S2). Therefore, whether the actions of BRD73879 that have been reported are due to RSK inhibition is not clear (Park and Cho, 2012; Theodosakis et al., 2017).

SC-1 (pluripotin) was identified in a cell-based screen and contains 3,4-dihydropyrimido (5,5-d)pyrimidine backbone (Mertins et al., 2013) (Figure 5E). SC-1 inhibits RSK1, RSK2 and RSK3 with an IC_50_ of −0.5, 2.5 and 3.3 μM, respectively, in an *in vitro* kinase assay but also numerous other targets (Supplementary Table S2) (Azhar et al., 2023).

## 4 Conclusion and future perspectives

RSK is associated with numerous disease pathologies but has primarily been studied in the context of cancer (Roskoski, 2019). The intense interest in RSK in cancer is due to the observations that the MAPK signaling pathway is a major oncogenic driver and that numerous cancers overexpress RSK (http://cancergenome.nih.gov/) (Figure 3). The mutation rate for individual RSK members is ≤2% in cancer with hot spots for RSK2, RSK3, and RSK4 occurring in the NTKD. Analysis of the mutations in the CTKD of RSK1 may alter protein stability but their effect on the NTKD catalytic activity was not described (Chikhale et al., 2023). RSK phosphorylates numerous substrates and the importance of any individual substrate to the transformation process is most likely dependent on cellular context. Despite the importance of RSK in cancer the development of specific RSK inhibitors has substantially lagged behind the efforts focused on other members of the MAPK signaling pathway. Importantly, RSK is being recognized as an important drug target in oncology and in support of this statement a RSK inhibitor, PMD-026, is in a phase 1/1b clinical trial. PMD-026 is reported to have a good safety profile in patients in contrast to MEK1/2 and ERK1/2 inhibitors (Kosnopfel et al., 2023). Results from the dose escalation portion of the trial found that PMD-026 extended progression free survival in heavily pre-treated patients with metastatic breast cancer (Beeram et al., 2021). These exciting results support the druggability of RSK and the continuation of efforts to develop additional RSK inhibitors.

Data obtained from the clinical trials using PMD-026 should provide information requiring the need for isoform specific inhibitors. The individual members of the RSK family contribute to differing homeostatic functions as identified by mutation and knockout of family members. Currently, the only isoform specific inhibitors are based on SL0101, which specifically inhibits RSK1 and RSK2 and trovafloxacin, which inhibits RSK4. Both of these inhibitors have shown efficacy in xenograft models. In cancer RSK1 and RSK2 regulate differing transcriptional programs in glioblastoma multiforme (Yang et al., 2022). Additionally, in lung cancer RSK1 appears to function as a tumor suppressor whereas RSK2 promoted metastasis (Lara et al., 2011). RSK4 is considered a tumor suppressor in ovarian cancer (Arechavaleta-Velasco et al., 2016), acute myeloid leukemia (Rafiee et al., 2016) and colorectal cancer (Ye et al., 2018). These results indicate that the development of isoform specific compounds may be necessary for RSK inhibitors to be successful in the clinic. The NTKDs are very similar to each other, which makes it difficult to identify isoform specific inhibitors (Figure 1). However, the exception may be RSK4, which appears to have an allosteric site in the NTKD. It is also possible that crystallization of the individual RSK isoforms may reveal binding pockets outside the NTKD that would inhibit kinase activity. Support for this hypothesis is provided by biochemical evidence obtained from an analogue of SL0101, which shows preferential binding to RSK2 *versus* RSK1(Wright et al., 2021). This analogue contains an n-propyl-carbamate at the 4” position of the rhamnose. Puzzlingly, based on the crystal structure of the RSK2 NTKD bound to SL0101 the n-propyl-carbamate would interact with the solvent. Therefore, to explain the preference for RSK2 *versus* RSK1 it seems reasonable to suggest that in the holokinase, the n-propyl-carbamate interacts with regions in the kinase which differ between RSK1 and RSK2. We speculate that these differences may be exploited for the development of isoform specific inhibitors.
